# Planetary health and environmental sustainability in African health professions education

**DOI:** 10.4102/phcfm.v15i1.3925

**Published:** 2023-02-21

**Authors:** James H. Irlam, Charlotte Scheerens, Bob Mash

**Affiliations:** 1 Department of Family, Community and Emergency Care, Faculty of Health Sciences, University of Cape Town, Cape Town, South Africa; 2 Department of Public Health and Primary Care, Faculty of Medicine, University of Ghent, Ghent, Belgium; 3 Department of Economics, Faculty of Medicine, University of Ghent, Ghent, Belgium; 4 Department of Family and Emergency Medicine, Faculty of Medicine and Health Sciences, Stellenbosch University, Cape Town, South Africa

**Keywords:** planetary health, sustainable healthcare, health professions education, environmental sustainability, climate change

## Abstract

CliMigHealth and the Education for Sustainable Healthcare (ESH) Special Interest Group of the Southern African Association of Health Educationalists (SAAHE) call for the urgent integration of planetary health (PH) and environmental sustainability into health professions curricula in Africa. Education on PH and sustainable healthcare develops much-needed health worker agency to address the connections between healthcare and PH. Faculties are urged to develop their own ‘net zero’ plans and to advocate for national and sub-national policies and practices that promote the Sustainable Development Goals (SDGs) and PH. National education bodies and health professional societies are urged to incentivise innovation in ESH and to provide discussion forums and resources to support the integration of PH into curricula.

**Contribution:**

This article provides a position statement for integrating planetary health and environmental sustainability into African health professions education curricula.

## Introduction

Education for sustainable healthcare (ESH) has been defined as the organisation of health professions education to develop knowledge, skills and attitudes about the interdependence of human health and ecosystems, including the effects of climate and environmental changes on health, and the impacts of health systems on the environment.^1^ Put simply, planetary health (PH) is the health of human civilisation and the state of the natural systems on which it depends.^2^

The 2021 AMEE *Consensus Statement on Planetary Health and Education for Sustainable Healthcare* is an important milestone in the development of ESH worldwide, which recognises the agency of health professionals to protect the planetary foundations of health and urges collective action: *Our health and well-being are dependent on a healthy planet. The window of opportunity to protect our ecosystems is fast disappearing, so urgent, collective, transdisciplinary action is required. The 2020s can be the decade in which we step up action on pressing issues such as a changing climate*. The consensus statement outlines the changes required in health professions education, approaches to achieve these changes, and a timeline for action linked to the Sustainable Development Goals (SDGs) by the year 2030.^1^

The AMEE call has since been amplified by others. The World Health Organization (WHO) *COP26 Special Report on Climate Change and Health*^3^ urged that health workers be trained to help build low-carbon and climate-resilient healthcare systems. The WHO-Civil Society Working Group to Advance Action on Health and Climate Change called on health education stakeholders to incorporate climate change into curricula for preparing health professionals ‘to recognize and address the health risks and impacts of climate change and to ensure functioning healthcare systems in a climate-changed future’.^4^ An ESH curriculum by the UK Medical Schools Council^5^ is among the latest additions to a growing global library of ESH resources.

In response to these calls, CliMigHealth and the ESH Special Interest Group of the Southern African Association of Health Educationalists (SAAHE) have launched the following *Position Statement on Integrating Planetary Health and Environmental Sustainabilit y into African Health Professions Education Curricula*.

## Position statement on integrating planetary health and environmental sustainability into African health professions education curricula

### Purpose

To call for enabling, accelerating and facilitating the urgent integration of planetary health (PH), including environmental sustainability, into health professions curricula in Africa, from undergraduate education to continuing professional development.

### Target audience

Health policy advisors; Civil Society Organisations (CSOs); Health professionals; Health professions educators in Africa.

### Background

Climate change is both a global health threat and public health opportunity for building low-carbon and climate-resilient healthcare systems and societies. Africa is highly vulnerable to climate change impacts, with serious consequences for health, and therefore urgently needs to raise climate change awareness, and to build capacity in mitigation, adaptation and advocacy.

The healthcare sector has a key role to play in meeting the global Sustainable Development Goals (SDGs), achieving zero carbon emissions by 2050, and in adapting and building resilience to growing climate change impacts. Health professionals have an ethical duty to lead in protecting public health and minimising the impact of health systems on the environment, and they are generally trusted as agents of change.

Faculties of health sciences and nursing and medical schools, therefore, need to train future health professionals to promote climate action and environmentally sustainable healthcare. Education on PH and environmentally sustainable healthcare develops their agency to address the connections between healthcare and planetary systems.

### Problem statement

Educational leadership on PH and education for sustainable healthcare (ESH) in Africa is lacking, however, and awareness is very low, especially in resource-constrained settings. Despite increasing interest in ESH in educational institutions, there is a general lack of meaningful action on environmental sustainability.

Planetary health and ESH are insufficiently integrated into African health professions curricula. Health professionals are consequently poorly equipped to act as agents for change on the scale and with the urgency required to respond to growing climate and environmental threats to public health.

### Recommendations

1. Share this position statement widely with public health agencies, CSOs, academic institutions and faculties of health sciences in Africa.
2. Urge faculties to measure their environmental footprints and to develop and publish plans to reach net zero by 2050 at the latest.
3. Urge faculties and educators to advocate for national and sub-national policies and practices that support the SDGs (especially SDG 3 Health and Wellbeing for All; SDG 4 Quality Education; SDG 13 Climate Action) and promote PH.
4. Encourage national education and accreditation bodies, and health professional societies, to offer incentives for innovation in ESH and PH (such as prizes for faculties with the best ‘net-zero’ plans; prizes for PH educators and champions; essay prizes for students; research grants and awards).
5. Provide an online platform for educators and students to share resources to support integration of PH into curricula in the African context, such as assessment rubrics, and tools for mapping and integrating PH into curricula.
6. Provide a platform for educators from different institutions to discuss ESH progress, challenges, approaches and leadership.
7. Host Special Interest Groups and events where educators can collaborate to develop new ESH and PH resources and means of assessment.
8. Monitor progress locally and regionally and disseminate examples of best practice.

This is a position statement on behalf of the ESH Special Interest Group of SAAHE and the CliMigHealth network. The views and opinions expressed in this article do not necessarily reflect the official policy or position of any other affiliated agency of the authors.

Refer to Table 1 for glossary of terms and explanations.

**TABLE 1:** Glossary of terms.

Terms	Explanation
Planetary health	The achievement of the highest attainable standard of health, well-being, and equity worldwide through judicious attention to the human systems – political, economic and social – that shape the future of humanity and the Earth’s natural systems that define the safe environmental limits within which humanity can flourish.^2^
Education for sustainable healthcare (ESH)	The process of equipping current and future health professionals with the knowledge, attitudes, confidence and capacity to provide environmentally sustainable services through health professional education.^6^ The UK’s Centre for Sustainable Healthcare used a consultation process to develop three Priority Learning Outcomes which encompass ESH^7^: Describe how the environment and human health interact at different levels. Demonstrate the knowledge and skills needed to improve the environmental sustainability of health systems. Discuss how the duty of a health worker to protect and promote health is shaped by the dependence of human health on the local and global environment.
Environmentally sustainable healthcare	Sustainable healthcare focuses on the improvement of health and better delivery of healthcare rather than late intervention in disease, with resulting benefits to patients and to the ecosystems on which human health depends, thus serving to provide high-quality healthcare now without compromising the ability to meet the health needs of the future.^8^
Environmental accountability	An environmentally accountable institution is both environmentally responsible and responsive: aware of global health challenges faced by society and recognises the environmental impact of its activities.^9^ The institution should also have policies in place to measure and reduce its environmental impact and work through education and research to promote sustainable healthcare.^10^
